# Reflections on my remote internship with Biology Open

**DOI:** 10.1242/bio.059726

**Published:** 2022-12-13

**Authors:** Sophie L. Johnson

**Affiliations:** Department of Plant Sciences, University of Oxford, Oxford OX1 3RB, UK

## Abstract

Sophie Johnson recounts her experience completing a remote internship at Biology Open, hosted by Editor-in-Chief Steve Kelly. Sophie is a third year BBSRC Doctoral Training Partnership (DTP) PhD student studying plant vein development at the University of Oxford. She was keen to get some experience in academic publishing and so carried out a PIPS (Professional Internships for PhD Students) placement working with Biology Open.

If you had asked me a year ago what a job in scientific publishing looked like I probably would have described (badly and in highly vague terms) the role of a journal editor. However, after a year working part-time as a publishing intern with Steve Kelly, Editor-in-Chief at Biology Open, I now appreciate the diversity of roles in the field. The internship has really opened my eyes to different career paths and has encouraged me to look at roles outside primary research.

As part of the internship, I was involved in the development of the ‘A Year at the Forefront…’ Review series and was lucky enough to follow the process from the conception of an initial idea to the publication of the first article. This gave me real insight into the time, effort and teamwork necessary to make such initiatives a reality. Publishing a new series starts a long time before any authors are involved. I worked with the Editor-in-Chief to draft the initial documents that defined the scope of the ‘A Year at the Forefront…’ series and enjoyed watching how feedback and discussions in the early stages of the project shaped the final product. A whole team of people then got to work to create website content, display items and marketing materials. The final test for the series was for me to write a Review using the new guidelines. I'd never written any kind of manuscript before so was a bit daunted by this, but the team at Biology Open and my PhD supervisor were very supportive. It was such a valuable experience, and I learnt a lot. The opportunity to complete a comprehensive literature review, synthesise primary research, and respond to reviewer comments means I am more confident about tackling my next publication. And despite how long it took me to write this reflection, I am more comfortable writing!

The ‘A Year at the Forefront…’ series forms part of Biology Open's programme of opportunities for early-career researchers (ECRs). I also contributed to their other initiatives for ECRs by inviting contributors for the Future Leader Reviews series. I particularly enjoyed this as it gave me an opportunity to sample some of the amazing research that is happening across a breadth of fields. A single day would see me exploring research from the molecular to the organismal, with stop-offs in development, immunology, neuroscience and even buzz pollination. My research also led me to numerous platforms dedicated to ECRs and gave me insight into some of the scientific communication and outreach work done by scientists around the world. I'm pretty shy, but even I couldn't fail to be inspired to engage more with the scientific community. I might not be ready for my first podcast appearance, but I will be signing up to more initiatives in the future.

My internship was remote and part-time, giving me the flexibility to continue working on my PhD. Despite this, every effort was made to include me in meetings and introduce me to the culture at The Company of Biologists. Before the internship, I had limited awareness of the publication process, Open Access publishing, and ethics issues, including identifying fraudulent research. Now I have a much better understanding of all the work that goes on behind the scenes to produce the journals I enjoy reading. Thank you to everyone at Biology Open and The Company of Biologists who provided me with this opportunity. If you're interested in a job in scientific publishing, I'd recommend an internship!

The Company of Biologists regularly hosts professional internships for ECRs. Each internship is carefully defined, both with the institute and the individual, and usually involves projects with our journal teams. Each year, a 12-week internship with a focus on data gathering and analysis is offered. From time to time, we offer additional internships, such as working on our blog, the Node. Hosted internships are funded through PhD programmes – created by funders to help further professional development. Find out more about working for us: https://www.biologists.com/about-us/work-for-us/.

